# Detecting overlapping protein complexes in weighted PPI network based on overlay network chain in quotient space

**DOI:** 10.1186/s12859-019-3256-9

**Published:** 2019-12-24

**Authors:** Jie Zhao, Xiujuan Lei

**Affiliations:** School of Computer Science, Shaanxi Normal University, Xi’an, 710119 Shaanxi China

**Keywords:** Protein complexes, Gene ontology, Quotient space, Granular computation, Clustering

## Abstract

**Background:**

Protein complexes are the cornerstones of many biological processes and gather them to form various types of molecular machinery that perform a vast array of biological functions. In fact, a protein may belong to multiple protein complexes. Most existing protein complex detection algorithms cannot reflect overlapping protein complexes. To solve this problem, a novel overlapping protein complexes identification algorithm is proposed.

**Results:**

In this paper, a new clustering algorithm based on overlay network chain in quotient space, marked as ONCQS, was proposed to detect overlapping protein complexes in weighted PPI networks. In the quotient space, a multilevel overlay network is constructed by using the maximal complete subgraph to mine overlapping protein complexes. The GO annotation data is used to weight the PPI network. According to the compatibility relation, the overlay network chain in quotient space was calculated. The protein complexes are contained in the last level of the overlay network. The experiments were carried out on four PPI databases, and compared ONCQS with five other state-of-the-art methods in the identification of protein complexes.

**Conclusions:**

We have applied ONCQS to four PPI databases DIP, Gavin, Krogan and MIPS, the results show that it is superior to other five existing algorithms MCODE, MCL, CORE, ClusterONE and COACH in detecting overlapping protein complexes.

## Introduction

Analyzing the mechanism of proteins is crucial for understanding the function of cell machinery and explaining biological processes [1]. Proteins often bind together to form complexes to carry out their biological functions [2, 3]. A protein complex is a molecular group of two or more functionally related proteins assembled via multiple protein interactions [4]. Detecting protein complexes has great significance in biology and proteomics [5]. In the early stage of protein complex research, the protein complexes were found mainly through biological experiments methods, such as RNA interference, conditional gene knockout, single gene knockout and Co-immunoprecipitation [6, 7]. However, these methods are costly and time-consuming.

The high throughput techniques have generated a large amount of protein related data. In 2001, Legrain et al. [8] described the protein-protein interactions (PPI) as an undirected graph G(*V*, *E*), where the point set *V* represents protein nodes and the edge set *E* represents protein-protein interactions. This idea transforms large-scale protein-protein interaction data into network structure, which triggered scholars to recognize protein complexes based on the topological properties of protein networks. In 2003, Bader and Hogue [9] proposed MCODE method which is a local-search method to detect protein complexes based on the proteins’ connectivity values in PPI network. In 2006, Gavin et al. [10] demonstrated that protein complexes was made up of core and additional attachment proteins or protein modules. According to the core-attachment structure of protein complexes, Leung et al. [11] designed CORE algorithm which calculated the *p-value* for all pairs of proteins to detect cores. Wu et al. [12] proposed COACH algorithm which detected dense subgraphs as cores. In 2009, Liu et al. [13] presented a method called CMC which identified protein complexes based on maximal cliques. In fact, a protein may belong to multiple protein complexes, and there may be overlaps between protein complexes. In 2012, NepusZ et al. developed a clustering algorithm ClusterONE [14] to detect overlapping protein complexes. Recently, attributed network embedding methods have be proved to be remarkably effective in generating vector representations for nodes in the network [15]. Xu et al. designed a method GANE to predict protein complexes based on Gene Ontology attributed network embedding [15].

Some classical clustering algorithms such as Markov Clustering (MCL) [16] and swarm intelligence optimization algorithm [17, 18] were also developed to detect protein complexes. Lei et al. [19] proposed F-MCL clustering model based on Markov clustering in which automatically adjusted the parameters by introducing the firefly algorithm. Wang et al. [4] developed a heuristic graph clustering algorithm called HGCA based on multiple topological characteristics.

In recent years, quotient space theory has been applied to cluster. Zhang [20] defined the fuzzy equivalence relation and stratified hierarchical structure, and established the fuzzy granular computing model in quotient space in order to solve the uncertain problem. Xu [21] proposed fuzzy clustering method based on Gaussian function. The method, with the nature of the distance metric spaces, merged the individual particles in information synthesis way for clustering results. Cluster analysis method [22] based on fuzzy similarity relations and normalized distance is proposed to solve data structure analysis of complex systems. The conclusion is suitable for the complicated systems.

In this study, a new clustering algorithm based on overlay network chain in quotient space, marked as ONCQS, was proposed to detect overlapping protein complexes in weighted PPI networks. Firstly, the GO annotation data is used to weight the PPI network. Then, the maximal complete subgraph of the PPI network is found. The maximal complete subgraph of the current network is regarded as the node in the next layer of network. According to the compatibility relation, the overlay network chain in quotient space is calculated, the protein complexes are contained in the last layer of the overlay network. The algorithm ONCQS is tested on four well-known PPI databases DIP [23], Gavin [10], Krogan [24] and MIPS [25]. The simulation results illustrate that ONCQS algorithm has a higher performance and outweighs than other five algorithms in mining protein complexes.

## Methods

### Constructing weighted PPI network

It is inaccurate to mine protein complexes directly in PPI networks because the data produced by high-throughput experiments contain a high rate of false positive and false negative interactions [26, 27].To address this problem, some scholars integrate protein biologic data such as gene expression data, subcellular localization data, GO annotation data [28, 29] to increase the reliability and accuracy of data. A protein complex is a group of two or more associated polypeptide chains. Different polypeptide chains may have same functions, so we integrate GO annotation data to measure the interactions. If two interacted proteins *v*_*i*_ and *v*_*j*_ have more common GO annotations, their functions are more similar and their interaction is believed to be more believable. The weight between protein *v*_*i*_ and *v*_*j*_ is defined as follows:

1 $$ {W}_{v_i,{v}_j}=\frac{\left|{GO}_{v_i}\cap {GO}_{v_j}\right|}{\mathit{\min}\left(\left|{GO}_{v_i}\right|,\left|{GO}_{v_j}\right|\right)} $$

where ${GO}_{v_i}$ and ${GO}_{v_j}$ are the GO annotation set of node *v*_*i*_ and *v*_*j*_ respectively, $\left|{GO}_{v_i}\cap {GO}_{v_j}\right|$ represents the number of the same annotation between ${GO}_{v_i}$ and ${GO}_{v_j}$. Our previous research shows that the ${W}_{v_i,{v}_j}$ value is greater than 0.6, and the effect is better [30]. If weight between protein *v*_*i*_ and *v*_*j*_ is less than 0.6, the interaction will be deleted in the PPI network. This preprocessing step can help us to filter out possible false positive interactions [31].

### Quotient space theory

Granular computing is a simulation of global analysis ability of human beings. One of the basic characteristics in human problem solving is the ability to conceptualize the world at different granularities and translate from one abstraction level to the others easily, deal with them hierarchically. Human beings can solve problems in different sizes of granularity spaces. Different levels represent different granularity.

There are three main theories of granular computing, granular computing based on fuzzy logic [32], granular computing based on rough set and granular computing based on quotient space. Granularity analysis is in fact to analyze the quotient set.

Triple structure (*X, F, T*) is used to represent the problem in the quotient space. Domain *X* refers to universe of discourse, *F* is the attribute set of *X*, *T* is the structure of *X*. Define a relation *R* for the universe of discourse *X*, construct corresponding quotient set [*X*], quotient attribute set [*F*], and quotient structure [*T*], and then define the granularity coefficient to study the quotient space([*X*], [*F*], [*T*]). The relation *R* can be equivalence relation or compatibility relation.

For the PPI network G, *G* = (*X*, *F*, *T*), domain *X* refers to the protein nodes in PPI network.

### Overlay network chain in quotient space

Given a network *G*, the maximum complete subgraph of the network is regarded as a cover according to the compatibility relation [33]. The pseudo code of the maximum complete subgraph algorithm is shown in Table 1.

**Table 1:**
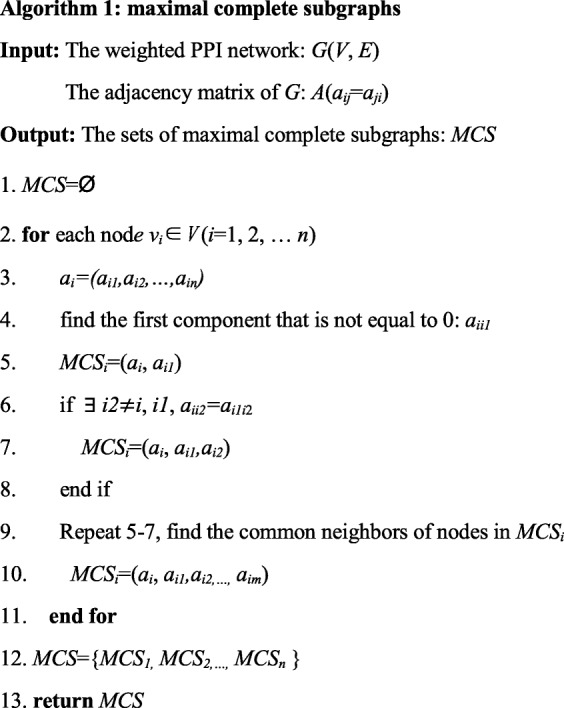
Pseudo code of maximum complete subgraph

**Fig. 1:**
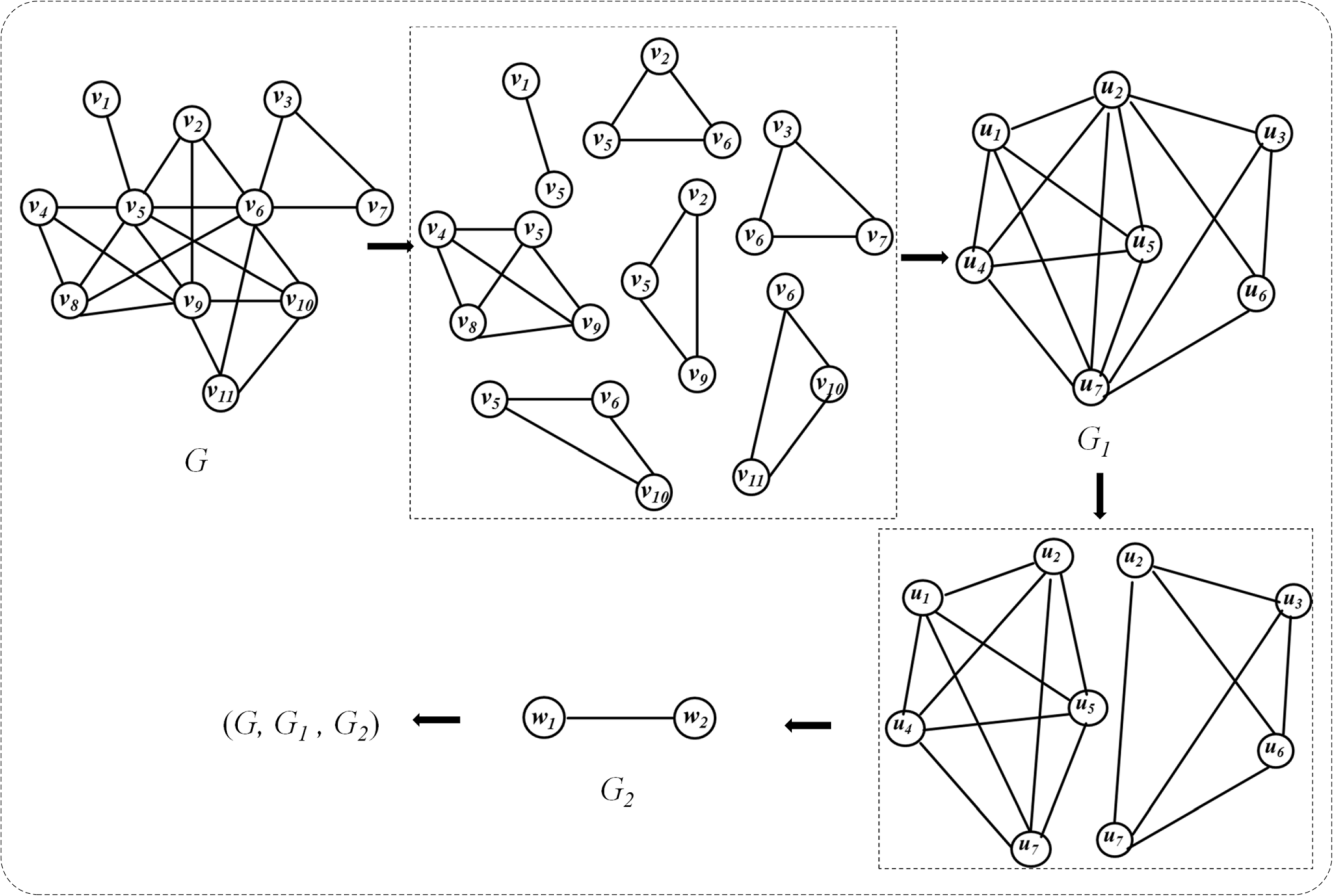
Construction of overlay network chain in quotient space

After the sets of all maximal complete subgraphs is solved. Then, maximal complete subgraphs are used as nodes, if two maximal complete subgraphs have common nodes, two corresponding nodes are defined to be connected, the new network constructed is called the 1^st^ level overlay network of *G* in quotient space, which is denoted as *G*_*1*_. Figure 1 illustrates the construction of overlay network chain in quotient space. The network *G* has 11 nodes (*v*_*1*_, *v*_*2*_, *v*_*3*_, *v*_*4*_, *v*_*5*_, *v*_*6*_, *v*_*7*_, *v*_*8*_, *v*_*9*_, *v*_*10*_, *v*_*11*_). There are 7 maximal complete subgraphs in the network *G*, so there are 7 nodes (*u*_*1*_, *u*_*2*_, *u*_*3*_, *u*_*4*_, *u*_*5*_, *u*_*6*_, *u*_*7*_) in the 1^st^ level overlay network *G*_*1*_. *u*_*1*_ represents (*v*_*1*_, *v*_*5*_), *u*_*2*_ represents (*v*_*2*_, *v*_*5*_, *v*_*6*_), *u*_*3*_ represents (*v*_*3*_, *v*_*6*_, *v*_*7*_), *u*_*1*_ and *u*_*2*_ has common nodes *v*_*5*_, *u*_*2*_ and *u*_*3*_ has common nodes *v*_*6*_, so *u*_*1*_ and *u*_*2*_ are connected in *G*_*1*_, *u*_*2*_ and *u*_*3*_ are connected in *G*_*1*_, *u*_*1*_ and *u*_*3*_ have no common nodes, and there is no connection between them in *G*_*1*_. Network *G*_*1*_ has two complete subgraphs, the 2nd level overlay network *G*_*2*_ has 2 nodes (*w*_*1*_, *w*_*2*_). *w*_*1*_ represents (*u*_*1*_, *u*_*2*_, *u*_*4*_, *u*_*5*_, *u*_*7*_), *w*_*2*_ represents (*u*_*2*_, *u*_*3*_, *u*_*6*_, *u*_*7*_), *w*_*1*_ and *w*_*2*_ has common nodes (*u*_*2*_, *u*_*7*_), so *w*_*1*_ and *w*_*2*_ are connected in *G*_*2*_. *G*_*1*_ and *G*_*2*_ are different levels of overlay network of *G*, (*G*, *G*_*1*_, *G*_*2*_) is called overlay network chain.

Assuming that *G*_*i*_ is the *i*^*th*^ level overlay network of *G*, and *G*_*i + 1*_ is the 1st level overlay network of *G*_*i*_, therefore, *G*_*i + 1*_ is the *(i + 1)*^*th*^ level overlay network of G. (*G*, *G*_*1*_, *G*_*2*_,…, *G*_*i*_) is called overlay network chain in quotient space [34].

### The ONCQS main algorithm

**Fig. 2:**
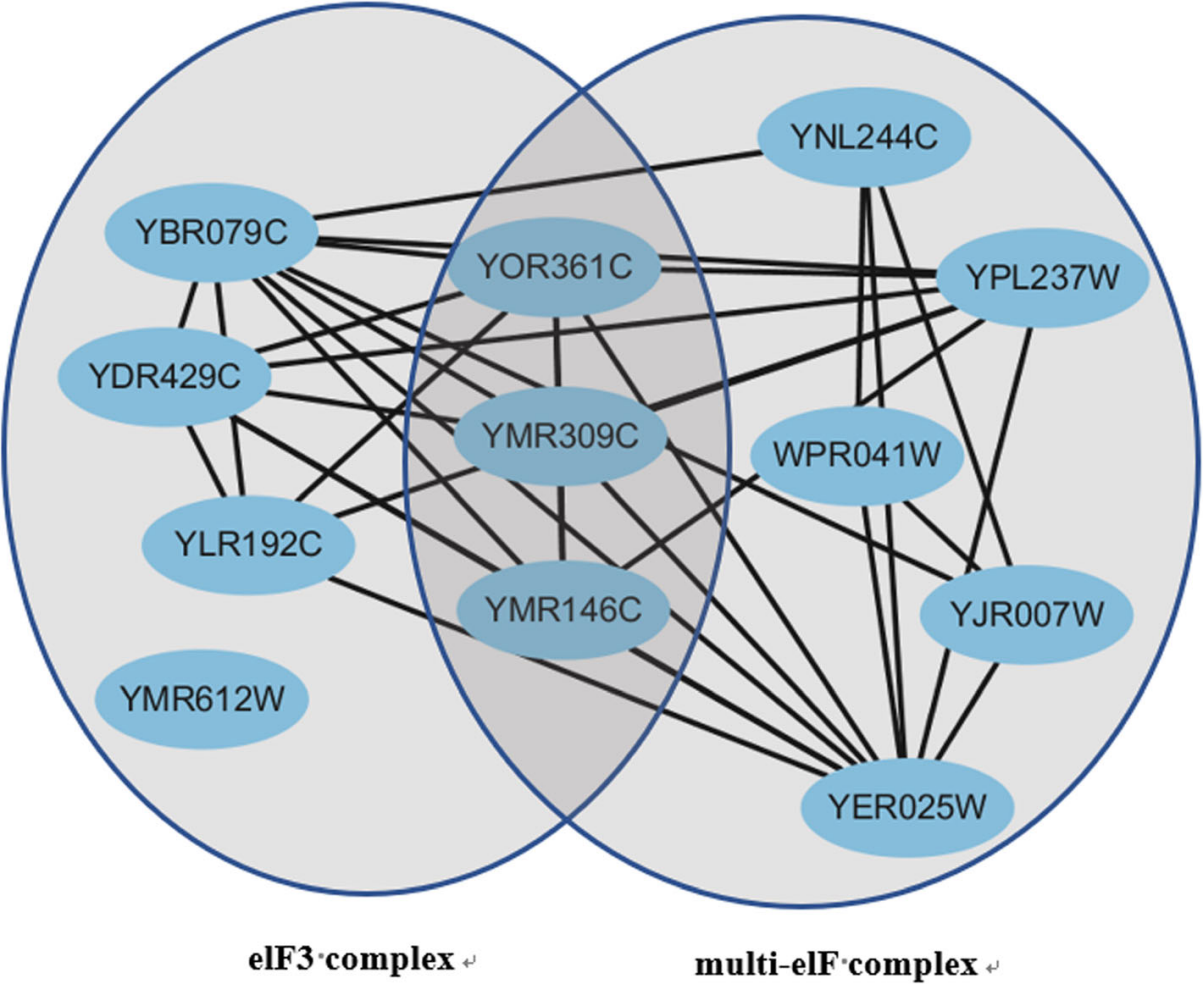
An example of overlapping protein complexes

A new clustering algorithm ONCQS is developed to detect overlapping protein complexes in weighted PPI network using overlay network chain in quotient space. A protein may belong to multiple protein complexes. As shown in Fig. 2, two protein complexes elF3 complex and multi-elF complex in the CYC2008 benchmark have three overlapped proteins.

In overlay network *G*_*i*_, each node represents a maximum complete subgraph of overlay network *G*_*i-1*_. There may be repeated points and edges between maximal complete subgraphs. The protein complexes are contained in the last level of the overlay network. Each point can be regarded as a complex. So overlapping protein complexes can be found by using covering network. As shown in Fig. 1, in *G*_*2*_, *w*_*1*_ represents (*v*_*1*_, *v*_*2*_, *v*_*4*_, *v*_*5*_, *v*_*6*_, *v*_*8*_, *v*_*9*_, *v*_*10*_), *w*_*2*_ represents (*v*_*2*_, *v*_*3*_, *v*_*5*_, *v*_*6*_, *v*_*7*_, *v*_*10*_, *v*_*11*_), they have four overlapped nodes.

In algorithm ONCQS, the static PPI network is usually described as an undirected graph G(*V*, *E*) which consists of a set of nodes *V* and a set of edges *E*, the nodes *V* represents the proteins and the edges *E* = {*e*(*v*_*i*_, *v*_*j*_)} is the set of edges connecting two proteins *v*_*i*_ and *v*_*j*_. First, we use GO annotation data to weight the PPI network, and then construct multilevel overlay network. In overlay network theory, if two maximal complete subgraphs have common nodes, two corresponding nodes are defined to be connected. However, in ONCQS algorithm, formula 2 is used to measure the similarity of two maximal complete subgraphs *mcs*_*i*_ and *msc*_*j*_.

2 $$ sim\left({mcs}_i,{mcs}_j\right)=\frac{\left|{mcs}_i\cap {mcs}_j\right|}{\left|{mcs}_i\cup {mcs}_j\right|} $$

where |*mcs*_*i*_ ∩ *mcs*_*j*_| is the number of the common nodes of *mcs*_*i*_ and *msc*_*j*_, |*mcs*_*i*_ ∪ *mcs*_*j*_| is the summation of the nodes of *mcs*_*i*_ and *msc*_*j.*_ Only when *sim*(*mcs*_*i*_, *mcs*_*j*_) is great than the granularity coefficient *gc*, two corresponding nodes are defined to be connected in the next level overlay network. In *i*^*th*^ level overlay network, if there is no maximal complete subgraph satisfying the similarity condition, the overlay network chain (*G*, *G*_*1*_*, G2,…, G*_*i*_) can be obtained. The pseudo code of the ONCQS algorithm is shown in Table 2.

**Table 2:**
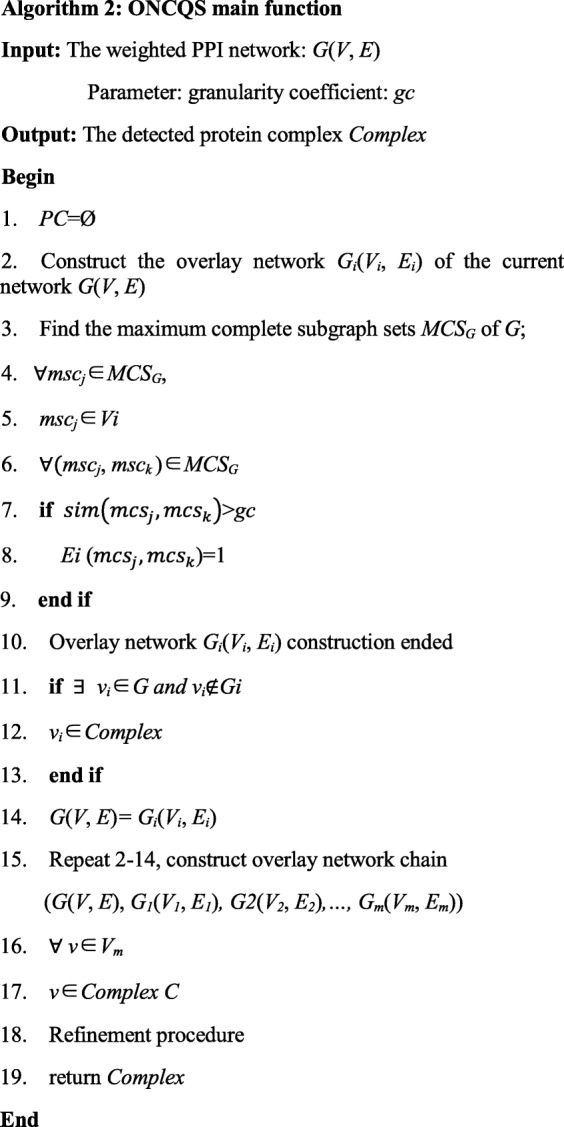
Pseudo code of the ONCQS algorithm

At this point, each node in *G*_*i*_ represents a protein complex. Each node represents a maximal complete subgraph, so the proteins in the subgraph have high similarity and the similarity between the subgraphs is poor.

## Results and discussion

The proposed ONCQS algorithm is implemented in Matlab R2015b and executed on a quad-core processor 3.30GHz PC with 8G RAM.

### Experimental data set

In this study, the developed methods and computational analysis were applied to four PPI network, including DIP [23], Gavin [10], Krogan [24] and MIPS [25]. All the data used in this study are *Saccharomyces cerevisiae* protein data.

Protein-protein interactions data: After removing the noise, the self-interactions and the repeated interactions, DIP dataset (version of 20160114) included 5028 proteins and 22,302 interactions, Gavin dataset consists of 1430 proteins and 6531 interactions, Krogan dataset consists of 2674 proteins and 7075 interactions, the MIPS dataset included 4546 proteins and 12,319 interactions.

Gene Ontology data: The *Saccharomyces cerevisiae* GO annotation data was extracted from GO-slims dataset. GO-slims data are cut-down version of the GO ontologies [31]. GO-slim data provide GO terms to explain gene product feature in biological process (BP), molecular function (MF), cellular component (CC). we used GO slims to annotate PPI data. There are 7014 proteins in the GO annotation data. Proteins with GO annotation data cover 98.23% of proteins in the DIP dataset, 100% of proteins in Gavin, 99.89% of proteins in Krogan, 99.16% of proteins in MIPS.

The standard protein complexes: CYC2008 [35] is used to evaluate clustering results of *Saccharomyces cerevisiae*, which includes 408 protein complexes. Detailed data intersection information of experimental data is shown in Table 3.

**Table 3:** The data information of the experimental data

Dataset	Number of node	Number of edge	Density	GO annotation data
DIP	5028	22,302	0.0018	4939 (98.23%)
Gavin	1430	6531	0.0064	1430 (100%)
Krogan	2674	7075	0.0020	2671 (99.89%)
MIPS	4546	12,319	0.0012	4508 (99.16%)

### Evaluation metrics

The overlapping score *OS* is used to evaluate the match quality of a predicted protein complex and standard protein complex.

3 $$ \mathrm{OS}\left( pc, sc\right)=\frac{{\left|{V}_{pc}\cap {V}_{sc}\right|}^2}{\mid {V}_{pc}\mid \times \mid {V}_{sc}\mid } $$

where *V*_*pc*_ and *V*_*sc*_ denote the node sets of predicted protein complex *pc* and standard protein complex *sc,* respectively. Usually we set the threshold for 0.2 [17]. If *OS*(*pc*, *sc*) is greater than 0.2, the predicted protein complex *pc* is considered to match standard protein complex *sc. OS* = 1 shows that the predicted protein complex is perfectly matched with the standard protein complex.

Three commonly used metrics *Precision, Recall* and *F-measure* are used to measure the efficiency of the proposed ONCQS algorithm and evaluate the performance of the clustering results.

The *Precision* denotes the accuracy of the predicted protein complexes matched by the standard protein complexes, defined as follows:

4 $$ Precision=\frac{\mid mpc\mid }{\mid pc\mid } $$

where ∣*pc*∣ represents the number of predicted protein complexes, ∣*mpc*∣ denotes the number of the predicted protein complexes matched by the standard protein complexes.

The *Recall* denotes the accuracy of the standard protein complexes matched by the predicted protein complexes, defined in the following eq. (5):

5 $$ Recall=\frac{\mid msc\mid }{\mid sc\mid } $$

where ∣*sc*∣ represents the number of the standard protein complexes, ∣*msc*∣ denotes the number of the standard protein complexes matched by the predicted protein complexes.

The *Precision* and *Recall* describe the accuracy of the algorithm from different aspects. In order to consider these two indicators synthetically, the *F-measure* is defined as the harmonic mean of *Precision* and *Recall. F-measure* is defined as follows:

6 $$ F- measure=\frac{2\times Precision\times Recall}{Precision+ Recall} $$

### Parameter analysis

The proposed algorithm ONCQS only has one parameter, granularity coefficient: *gc*. In overlay network, the similarity of two maximal complete subgraphs is greater than *gc*, we consider them connected in the next level overlay network. If the value of *gc* is too small, the complexity of algorithm will increase. On the contrary, if the value of *gc* is too large, the accuracy of the algorithm will decrease. It is significant to select the appropriate value of *gc*.

The experiments on four PPI databases with *gc* from 0.1 to 0.9 were carried out to verify the influence of parameter *gc*. The results are shown in Table 4. where *PC* is the total number of predicted protein complexes, *Perfect* is the count of predicted protein complexes and standard complexes are perfectly matched, *OS*(*pc*, *sc*) = 1. *AS* represents the average size of the predicted protein complexes.

**Table 4:** Influence of parameters *gc*

Dataset	*gc*	*Precision*	*Recall*	*F-measure*	*PC*	*Perfect*	*AS*
DIP	0.1	0.4199	0.7108	0.5279	874	62	5.18
	0.2	0.4011	0.7206	0.5153	945	60	4.79
	0.3	0.3571	0.7402	0.4818	1095	69	3.70
	0.4	0.3561	0.8260	0.4976	1640	103	2.67
	0.5	0.3521	0.8284	0.4942	1667	104	2.60
	0.6	0.3470	0.8211	0.4878	1781	105	2.53
	0.7	0.3499	0.8186	0.4902	1832	103	2.54
	0.8	0.3530	0.8186	0.4933	1844	102	2.56
	0.9	0.3530	0.8186	0.4933	1844	102	2.56
Gavin	0.1	0.6581	0.4167	0.5103	310	38	7.99
	0.2	0.6085	0.4265	0.5015	355	39	6.63
	0.3	0.5630	0.4363	0.4916	405	41	5.13
	0.4	0.5124	0.4510	0.4797	525	49	3.98
	0.5	0.4973	0.4534	0.4743	553	50	3.73
	0.6	0.4879	0.4461	0.4661	621	50	3.46
	0.7	0.4910	0.4436	0.4661	664	46	3.43
	0.8	0.4927	0.4436	0.4669	684	46	3.47
	0.9	0.4949	0.4436	0.4679	687	46	3.49
Krogan	0.1	0.5856	0.5956	0.5906	473	68	4.51
	0.2	0.5658	0.5980	0.5815	509	67	4.27
	0.3	0.5401	0.5980	0.5676	561	68	3.60
	0.4	0.4888	0.6422	0.5551	759	80	2.86
	0.5	0.2728	0.7230	0.3962	780	81	2.82
	0.6	0.3095	0.7230	0.4335	835	83	2.77
	0.7	0.2984	0.7230	0.4225	858	78	2.76
	0.8	0.3090	0.6005	0.4080	868	79	2.79
	0.9	0.4989	0.6471	0.5634	870	79	2.81
MIPS	0.1	0.3784	0.5735	0.4559	703	46	3.95
	0.2	0.3689	0.5760	0.4498	721	47	3.78
	0.3	0.3375	0.5980	0.4315	803	54	3.10
	0.4	0.3231	0.6765	0.4373	1173	72	2.33
	0.5	0.3238	0.6765	0.4379	1186	71	2.32
	0.6	0.3288	0.6691	0.4409	1244	69	2.31
	0.7	0.3299	0.6642	0.4408	1255	67	2.32
	0.8	0.3315	0.6642	0.4423	1258	67	2.32
	0.9	0.3315	0.6642	0.4423	1258	67	2.33

*F-measure* reflects the effectiveness of the algorithm, and *Perfect* reflects the accuracy of the algorithm. In order to comprehensively consider the impact of *gc* on the performance of the algorithm, we performed min-max normalization on *F-measure* and *Perfect*. The parameter *F* is defined as the harmonic mean of *F-measure* and *Perfect*, as shown in eq. (9).

7 $$ NFmeasure=\frac{F- meausre-\min \left(F- measure\right)}{\max \left(F- measure\right)-\min \left(F- measure\right)} $$

8 $$ NPerfect=\frac{Perfect-\min (Perfect)}{\max (Perfect)-\min (Perfect)} $$

9 $$ F=\frac{NFmeasure+ NPerfect}{2} $$

**Fig. 3:**
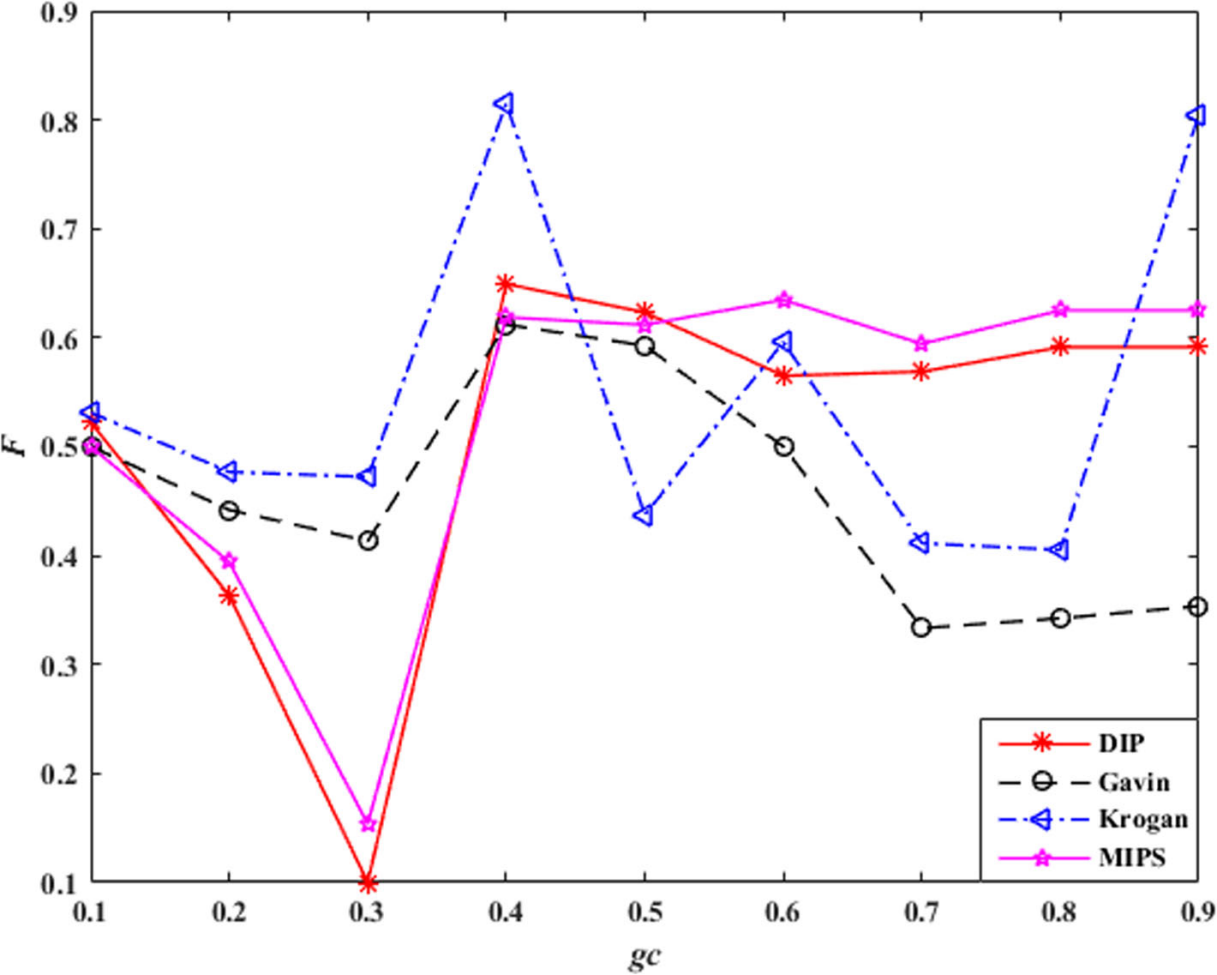
Influence of parameters *gc*

The influence of parameters *gc* is shown in Fig. 3. *F* value gets the best value when *gc* equals 0.4 in DIP, Gavin and Krogan. When *gc* is greater than 0.4 the *F* value will rise tends to be stable in MIPS. So set *gc* for 0.4 in this study.

### Comparison based on precision, recall and F-measure

**Fig. 4:**
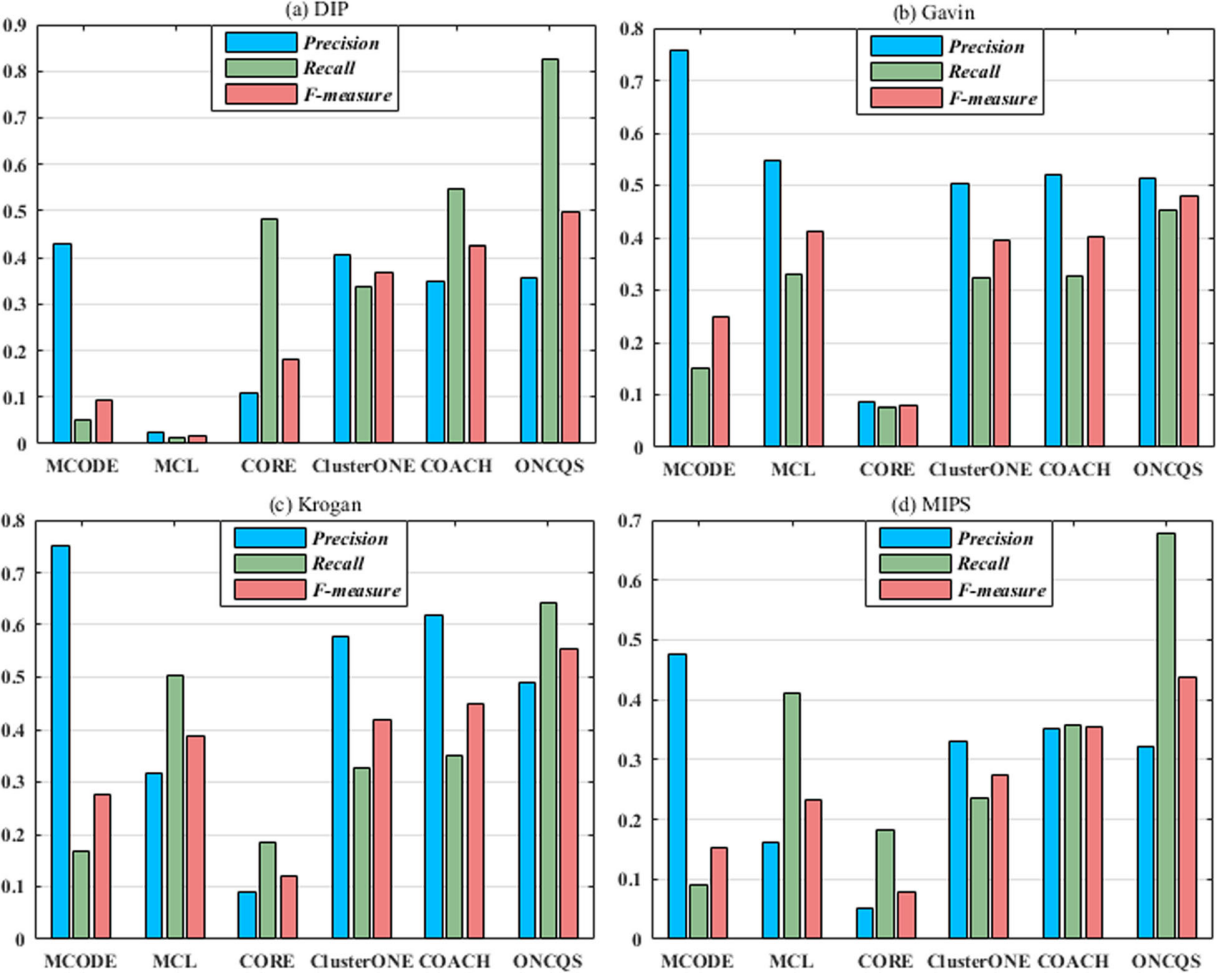
The performance comparisons of various algorithms on four datasets, the blue bar represents Precision, the green bar represents Recall, the red bar represents F-measure. (**a**) DIP (**b**) Gavin (**c**) Krogan (**d**) MIPS

The performance of ONCQS is compared with five other state-of-the-art protein complex prediction algorithms: MCODE, MCL, CORE, ClusterONE and COACH. The MCODE and ClusterONE are run using Cytoscape [36] and the parameters are set to the default setting. Figure 4 depicts the *Precision, Recall and F-measure* of each algorithm on four datasets. As shown in Fig. 4, it is obvious that the *Recall* and *F-measure* value of our method is much more excellent than other methods on four datasets. It indicates that ONCQS algorithm can detect protein complexes more accurately. In Fig. 4a DIP dataset, the ONCQS achieved *Precision, Recall and F-measure* values of 0.3561, 0.8260 and 0.4976, respectively. The other methods MCODE, MCL, CORE, ClusterONE and COACH achieved *F-measure* values 0.0919, 0.0168, 0.1794, 0.3690 and 0.4270. In Fig. 4b Gavin dataset, the ONCQS achieved the highest *Recall* 0.4510 *and F-measure* 0.4797*.* In Fig. 4c Krogan dataset, the ONCQS achieved the highest *Recall* 0.6422 *and F-measure* 0.5551*,* which obviously outperforms other methods. In Fig. 4d, the methods MCODE,MCL, CORE, ClusterONE, COACH and ONCQS achieved *F-measure* values 0.1524, 0.2321, 0.0796, 0.2755, 0.3548 and 0.4373. Table 5 depicts the *PC, Perfect and AS* of each algorithm on four datasets. Obviously, the algorithm ONCQS can mine the protein complex more accurately, and the *perfect* value is much higher than other algorithms.

**Table 5:** The performance comparison of several typical algorithms on four datasets

Algorithms	DIP			Gavin			Krogan			MIPS		
	*PC*	*Perfect*	*AS*	*PC*	*Perfect*	*AS*	*PC*	*Perfect*	*AS*	*PC*	*Perfect*	*AS*
MCODE	49	1	16.73	66	8	9.12	76	11	7.21	63	3	8.33
MCL	189	0	3.76	217	20	6.83	550	17	4.63	922	12	4.67
CORE	1707	6	3.01	294	0	2.58	820	0	2.32	1745	0	2.18
ClusterONE	372	6	4.94	243	13	6.92	241	12	5.26	295	3	4.24
COACH	899	16	8.90	321	12	10.18	355	17	7.55	489	9	10.31
ONCQS	1640	103	2.67	525	49	3.98	759	80	2.86	1173	72	2.34

### Comparison with standard complexes

**Fig. 5:**
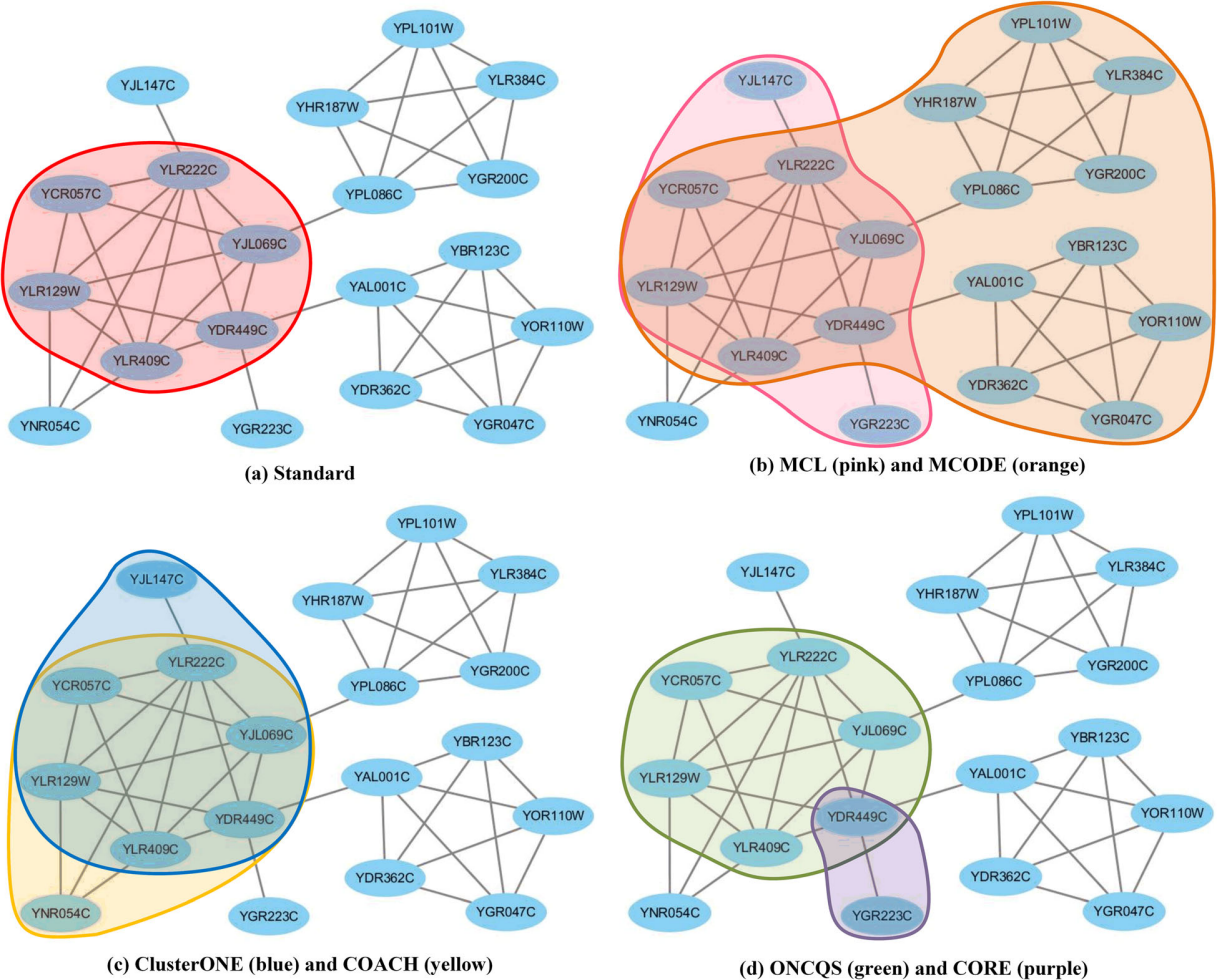
Visualization of the 379^th^ standard protein complex of Krogan. (**a**) Standard (red area) (**b**) MCL (pink area) and MCODE (orange area) (**c**) ClusterONE (blue area) and COACH (yellow area) (**d**) ONCQS (green area) and CORE (purple area)

In order to show the experimental results more clearly, we visualized the 379^th^ standard protein complex of CYC2008 “UTP B complex” and the corresponding mining results of 6 algorithm on Krogan dataset in Fig. 5. As shown in Fig. 5a, the standard protein complex is bound together by 6 proteins. Figure 5b shows the results of MCL and MCODE, the pink area is the result of the MCL algorithm, and the orange area is the result of MCODE. MCL algorithm has 2 proteins that are incorrect predictions. MCODE predicts three closely connected subgraphs into a protein complex. Figure 5c shows the results of ClusterONE and COACH, the blue area is the result of the ClusterONE algorithm, and the yellow area is the result of COACH. Both ClusterONE and COACH algorithms have a mispredicted protein. In Fig. 5d, green area and purple area are the results of ONCQS and CORE respectively. ONCQS correctly found 6 proteins. Other algorithms have erroneous prediction of proteins.

### Compare the ability to mine overlapping protein complexes

Individual proteins can participate in the formation of a variety of different protein complexes, different complexes perform different functions. There are overlaps between protein complexes. ONCQS method is proposed to mine overlapping protein complexes. The standard protein complexes in the CYC2008 database contain many overlapping protein complexes. Figure 2 shows a pair of overlapping protein complexes elF3 complex and multi-elF complex. We analyzed the matching of the six algorithms in four databases to these two complexes. The elF3 complex and multi-elF complex were recorded as *sc1* and *sc2*. Their complexes information is listed in Table 6.

**Table 6:** The complexes information of elF3 complex and multi-elF complex

elF3 complex *(sc1)*	multi-elF complex *(sc2)*
YMR012W YLR192C YMR309C YOR361C YBR079C YMR146C YDR429C	YER025W YMR309C YOR361C YNL244C YJR007W YPL237W YMR146C YPR041W

The elF3 complex contains seven proteins, multi-elF complex contains eight proteins, three of which are common. Then we analyze the clustering results of the 6 algorithms in four databases respectively. Similarly, only when the overlapping score is greater than 0.2, the matching is considered successful, and when there are multiple successful matches, the maximum overlapping score is obtained. The results of the 6 algorithms in DIP, Gavin, Krogan and MIPS are shown in Tables 7, 8, 9 and 10 respectively. Where *pc1* represents the predicted complex that matches elF3 complex (*sc1)*, *pc2* represents the predicted complex that matches multi-elF complex (*sc2)*. The boldface indicates that the proteins are predicted correctly.

**Table 7:** The performance comparison of mining overlapping proteins in DIP

Algorithm	Predicted elF3 complex *(pc1)*	*OS(pc1,sc1)*	Predicted *multi-elF complex (pc2)*	*OS(pc2,sc2)*
MCODE	–	–	–	–
MCL	–	–	–	–
CORE	**YMR146C YDR429C YBR079C**	0.4286	–	–
ClusterONE	YPR041W **YDR429C YBR079C YMR309C YMR146C** YPL001W **YOR361C** YDR091C **YLR192C** YPL105C	0.5143	–	–
COACH	**YDR429C YBR079C YMR146C YMR309C** YNL244C **YOR361C** YPR041W YPR086W **YLR192C**	0.5714	–	–
ONCQS	**YBR079C YDR429C YLR192C YMR146C YMR309C** YNL244C **YOR361C** YPR041W	0.6429	YBR079C **YJR007W YPL237W YPR041W**	0.2813

**Table 8:** The performance comparison of mining overlapping proteins in Gavin

Algorithm	Predicted elF3 complex *(pc1)*	*OS(pc1,sc1)*	Predicted *multi-elF complex (pc2)*	*OS(pc2,sc2)*
MCODE	**YDR429C YBR079C YMR309C**	0.4286	–	–
MCL	**YBR079C** YDR091C **YDR429C YLR192C YMR309C** YPR041W **YOR361C YMR146C** YNL244C YNL096C	0.5143	–	–
CORE	–	–	–	–
ClusterONE	**YMR309C YMR146C** YOR096W YOR204W **YOR361C** YPR041W YNL096C YNL244C **YBR079C** YDR091C	0.4286	–	–
COACH	YNL096C YPR041W **YOR361C YMR146C** YOR204W YAL035W **YBR079C YDR429C YLR192C YMR309C** YOL120C YJR123W	0.4286	**YNL244C** YDR429C **YOR361C YMR146C** YOR204W YAL035W YBR079C YLR192C **YMR309C YPR041W** YJL190C YBL072C YJR123W	0.2404
ONCQS	YAL035W **YBR079C YDR429C YLR192C YMR309C** YPR041W **YOR361C YMR146C**	0.6429	–	–

**Table 9:** The performance comparison of mining overlapping proteins in Krogan

Algorithm	Predicted elF3 complex *(pc1)*	*OS(pc1,sc1)*	Predicted *multi-elF complex (pc2)*	*OS(pc2,sc2)*
MCODE	–	–	–	–
MCL	YBR065C **YBR079C** YCR060W YDR047W YDR408C **YDR429C** YGL016W YHR034C **YMR309C YOR361C** YPR041W	0.2078	–	–
CORE	–	–	–	–
ClusterONE	**YOR361C** YER025W **YMR309C YBR079C** YPL105C **YMR146C** YBR065C **YDR429C** YPR041W	0.3968	–	–
COACH	**YMR146C YMR309C YDR429C** YBR065C **YBR079C YOR361C** YPR041W	0.5102	**YJR007W** YBR079C **YMR146C YMR309C YOR361C YPR041W YER025W** YDR429C	0.5625
ONCQS	**YBR079C YDR429C YMR146C YMR309C YOR361C**	0.7143	YBR079C **YER025W YJR007W YOR361C YPR041W**	0.4000

**Table 10:** The performance comparison of mining overlapping proteins in MIPS

Algorithm	Predicted elF3 complex *(pc1)*	*OS(pc1,sc1)*	Predicted *multi-elF complex (pc2)*	*OS(pc2,sc2)*
MCODE	–	–	–	–
MCL	**YBR079C YDR429C YMR146C YMR309C** YNL244C **YOR361C** YPL105C YPR041W	0.4464	–	–
CORE	–	–	–	–
ClusterONE	–	–	**YPR041W YNL244C YOR361C YMR146C YMR309C** YBR079C	0.5208
COACH	**YMR146C YOR361C YDR429C YMR309C** YPL105C	0.4571	**YMR309C YOR361C YPR041W** YBR079C **YMR146C YNL244C**	0.5208
ONCQS	**YDR429C YMR146C YOR361C**	0.4286	YBR079C **YMR309C YNL244C YOR361C YPR041W**	0.4000

As shown in Tables 7, 8, 9 and 10, MCODE, MCL, CORE and ClusterONE cannot detect overlapping protein complexes. MCODE and CORE failed to dig out complexes that match *sc1* and *sc2* respectively. COACH can dig out protein complexes that match *sc1* and *sc2*, the accuracy is not as good as ONCQS. ONCQS achieved the best performance in identifying overlapping protein complexes. Both CluterONE and COACH algorithms are proposed for mining overlapping protein complexes. In this case, ClusterONE cannot detect overlapping protein complexes, and the performance of COACH is poor. This further shows that it is meaningful to design efficient and accurate algorithms to mine overlapping protein complexes. ONCQS combines GO functional annotation information, which can improve the accuracy of the algorithm.

## Conclusion

Protein complexes are involved in multiple biological processes, and thus the detection of protein complexes is essential to understand cellular mechanisms. At the same time, there is overlap between protein complexes. This paper proposes a new algorithm ONCQS to identify overlapping protein complexes based on overlay network chain in quotient space. Combining the network properties of protein interaction networks with the biological properties of proteins, protein complexes are seen as nodes in the overlay network. Build an overlay network chain to mine protein complexes. Compared with the other competing clustering methods, ONCQS can effectively identify the overlapping protein complexes and has higher precision and accuracy.

## Data Availability

The methods MCODE and ClusterONE are run using Cytoscape and the parameters are set to the default setting. GO-slims data available at https://downloads.yeastgenome.org/curation/literature/go_slim_mapping.tab.

## Abbreviations

GO: Gene ontology
MCL: Markov clustering
ONCQS: Overlay network chain in quotient space
OS: Overlapping score
PPI: Protein-protein interaction
